# Hydroxymethylbutenyl diphosphate accumulation reveals MEP pathway regulation for high CO_2_-induced suppression of isoprene emission

**DOI:** 10.1073/pnas.2309536120

**Published:** 2023-10-02

**Authors:** Abira Sahu, Mohammad Golam Mostofa, Sarathi M. Weraduwage, Thomas D. Sharkey

**Affiliations:** ^a^Department of Energy Plant Research Laboratory, Michigan State University, East Lansing 48824, MI; ^b^Plant Resilience Institute, Michigan State University, East Lansing 48824, MI; ^c^Department of Biochemistry and Molecular Biology, Michigan State University, East Lansing 48824, MI; ^d^Department of Biology and Biochemistry, Bishop’s University, Sherbrooke JIE0L3, QC, Canada

**Keywords:** isoprene, chloroplast, elevated CO_2_, MEP pathway

## Abstract

Isoprene is emitted by some plants and is the most abundant biogenic hydrocarbon entering the atmosphere. Multiple studies have elucidated protective roles of isoprene against several environmental stresses, including high temperature, excessive ozone, and herbivory attack. However, isoprene emission adversely affects atmospheric chemistry by contributing to ozone production and aerosol formation. Thus, understanding the regulation of isoprene emission in response to varying environmental conditions, for example, elevated CO_2_, is critical to comprehend how plants will respond to climate change. Isoprene emission decreases with increasing CO_2_ concentration; however, the underlying mechanism of this response is currently unknown. We demonstrated that high-CO_2_-mediated suppression of isoprene emission is independent of photosynthesis and light intensity, but it is reduced with increasing temperature. Furthermore, we measured methylerythritol 4-phosphate (MEP) pathway metabolites in poplar leaves harvested at ambient and high CO_2_ to identify why isoprene emission is reduced under high CO_2_. We found that hydroxymethylbutenyl diphosphate (HMBDP) was increased and dimethylallyl diphosphate (DMADP) decreased at high CO_2._ This implies that high CO_2_ impeded the conversion of HMBDP to DMADP, possibly through the inhibition of HMBDP reductase activity, resulting in reduced isoprene emission. We further demonstrated that although this phenomenon appears similar to abscisic acid (ABA)-dependent stomatal regulation, it is unrelated as ABA treatment did not alter the effect of elevated CO_2_ on the suppression of isoprene emission. Thus, this study provides a comprehensive understanding of the regulation of the MEP pathway and isoprene emission in the face of increasing CO_2_.

## Significance

Isoprene has significant impacts on air quality and plant health. Because isoprene emission varies with changes in environmental conditions like light, temperature, and CO_2,_ a mechanistic understanding of the regulation in the face of climate change is essential to predict future isoprene emissions and its effect on the climate. In this study, we characterized CO_2_ responsiveness of isoprene at varying light and temperature. We also showed that an increase in upstream precursors but reduction in the immediate precursor of isoprene causes isoprene to decline, indicating an inhibition of a specific enzyme activity at high CO_2_. We further demonstrated that high CO_2_-mediated suppression of isoprene is independent of the stomatal signaling pathway.

Isoprene (C_5_H_8_, 2-methyl 1,3-butadiene) is a highly reactive, volatile hydrocarbon emitted by various plant species (1, 2). Isoprene accounts for more than half of the total amount of nonmethane biogenic volatile organic compounds emitted to the biosphere (3). In the presence of high level of atmospheric nitrogen oxides, one isoprene molecule can contribute to the production of multiple ozone molecules (4). In addition, isoprene is associated with the formation of aerosols, causing appearance of blue haze in the atmosphere (5). According to one estimate, isoprene accounts for nearly 55% of total secondary aerosol production in the eastern United States (6). Therefore, isoprene has significant impacts on tropospheric chemistry by contributing to ozone and secondary aerosol formation and increasing the lifetime of methane (7). Hence, it is crucial to comprehend the physiological mechanisms regulating isoprene emission from plants so that we can predict the effect of isoprene on future atmospheric conditions and how plants will respond to climate changes, such as increasing temperatures and CO_2_ concentrations.

In plants, isoprene synthesis begins with the methylerythritol-4-phosphate (MEP) pathway (8, 9). Carbon required for the synthesis of 1-deoxy-d-xylulose-5-phosphate (DXP), the first product of the MEP pathway, comes predominantly from the Calvin–Benson cycle (10).This pathway is also dependent on the photosynthetic electron transport chain for the supply of cytidine 5’-triphosphate (CTP), adenosine 5’-triphosphate (ATP), NADPH, and ferredoxin. Isoprene is synthesized from dimethylallyl diphosphate (DMADP) by isoprene synthase.

The rate of isoprene emission can vary depending on various environmental factors, including light, temperature, and CO_2_. Isoprene emission is light dependent (11, 12), and the light response is similar to that of photosynthesis except that isoprene emission often continues to increase with increasing illumination even after photosynthesis reaches saturation (13). Isoprene decreases immediately after lights are turned off, indicating the dependence of this phenomenon on the availability of NADPH, ATP, CTP, and ferredoxin from the photosynthetic electron transport chain. Isoprene emission is also affected by temperature variations (12, 14). High temperature leads to increased rates of isoprene emission from plants in both greenhouse and natural settings (14). Isoprene emission also responds to rapid temperature fluctuations (15). Besides light and temperature, CO_2_ is another well-studied environmental factor that substantially impacts isoprene emission from plants. In the presence of a low level of O_2_, isoprene emission decreases prominently with an increase in the CO_2_ level (12, 16). However, growing plants in high CO_2_ can affect isoprene emission differently depending on the type of plant species. For example, the rate of isoprene emission declines in aspen, whereas oaks emit more isoprene when they are grown in high CO_2_ environment (17). Since both CO_2_ levels and temperature are currently on the rise worldwide (18), many models have been created to predict the effect of these two parameters, alone or in combination, on future isoprene emission. Some of these models suggest an increase in isoprene emission by 25 to 75% in the 21st century (19–21). Based on an IPCC climate model (800 ppm CO_2_ and 33 °C), Lantz et al. (22) predicted that global isoprene emission could increase by as much as 50% by the year 2100 because the effect of high temperature would exceed the inhibition by elevated CO_2_.

Multiple studies have been conducted to identify the mechanism behind the high CO_2_-mediated inhibition of isoprene emission (22–24). Since isoprene emission is reduced within a few minutes of high CO_2_ treatment, changes in gene expression and protein levels are unlikely to explain this reduction. One of the earliest hypotheses put forward was that an increase in CO_2_ concentration stimulates the activity of phosphoenolpyruvate carboxylase (PEPC), leading to a reduction in cytosolic PEP, limiting the availability of pyruvate for the MEP pathway (23). However, inhibition of isoprene emission was not affected at elevated CO_2_ upon feeding hybrid poplar leaves with PEPC inhibitors (25). Moreover, PEPC activity was shown to decrease at high CO_2_ using stable isotope labeling (26). An alternative hypothesis is that isoprene emission is dependent on the availability of energy equivalents ATP and NADPH (24). ATP and NADPH levels are reduced during feedback inhibition of photosynthesis by high CO_2_ due to triose phosphate utilization (TPU) limitation of photosynthesis (27, 28), which could result in lower DMADP levels, reducing the rate of isoprene emission. This is supported by multiple studies showing that isoprene emission is correlated with the DMADP levels in plant tissues (29–31). However, Lantz et al. (22) demonstrated that suppression of isoprene emission at high CO_2_ is not correlated with TPU limitation. They also suggested that this phenomenon is independent of photosystem (PS)I, PSII, and ATP synthase energetics. Therefore, the underlying mechanism that causes the decrease of isoprene emission at high CO_2_ is not clearly understood.

We investigated the effect of light and temperature on the suppression of isoprene emission at elevated CO_2_ using gas exchange methods. We found that the CO_2_-mediated inhibition of isoprene emission is less at high temperature. Then, we used targeted metabolomics of leaves sampled at 41 Pa or 78 Pa CO_2_. We found one specific step in the MEP pathway that is inhibited by high CO_2_. Stomatal conductance declines at high CO_2_, especially in the presence of abscisic acid (ABA), so we tested the effect of ABA on isoprene emission, but ABA did not affect isoprene emission or the response of isoprene emission to CO_2_.

## Results

### Isoprene Emission Decreases with Increasing CO_2_ Level and Is Independent of Photosynthesis.

**Fig. 1.**
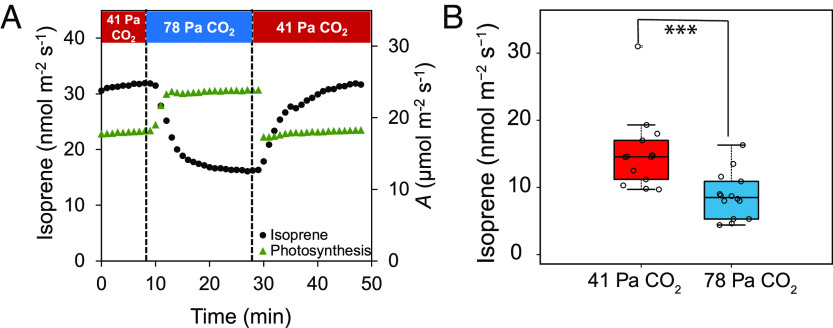
Effect of 41 Pa and 78 Pa CO_2_ on isoprene emission and photosynthesis in poplar leaves. (*A*) Time course of isoprene emission and photosynthesis as the CO_2_ level is switched between 41 Pa and 78 Pa in a poplar leaf. (*B*) Isoprene emission recorded in poplar leaves (*n* = 14) after they reached a stable value at 41 and 78 Pa CO_2_. Asterisks indicate significant decline in isoprene emission at 78 Pa CO_2_ compared with ambient CO_2_ (*P* < 0.001; Student’s *t* test). Whiskers of the box plots represent 95% CI.

Photosynthesis and isoprene emission from leaves were allowed to stabilize at 41 Pa CO_2_, 1,000 μmol m^−2^ s^−1^ light, and 30 °C, and then, the partial pressure of CO_2_ was increased to 78 Pa. Isoprene emission started to decline within 1 min of switching to 78 Pa CO_2_ and kept decreasing over time until it stabilized after 20 min of exposure to high CO_2_ (Fig. 1*A*). The average decrease in isoprene emission after switching from 41 to 78 Pa CO_2_ was 42 ± 12% (Fig. 1*B*). Isoprene increased upon returning to 41 Pa CO_2_ and stabilized near to the initial value before high CO_2_ treatment. Assimilation rates increased by 49 ± 18% (*SI Appendix*, Fig. S1*A*), whereas stomatal conductance did not show any significant change under these conditions (*SI Appendix*, Fig. S1*B*). As CO_2_ partial pressure was switched from 41 Pa to 78 Pa, photosynthesis increased as quickly as could be detected (within <1 min), whereas isoprene emission declined slowly over a course of 15 min (Fig. 1*A*). The initial phase of the isoprene decline followed first-order kinetics with a half-life of 6.1 ± 2 min.

### Effect of Varying Light Intensity on Suppression of Isoprene Emission at High CO_2_.

**Fig. 2.**
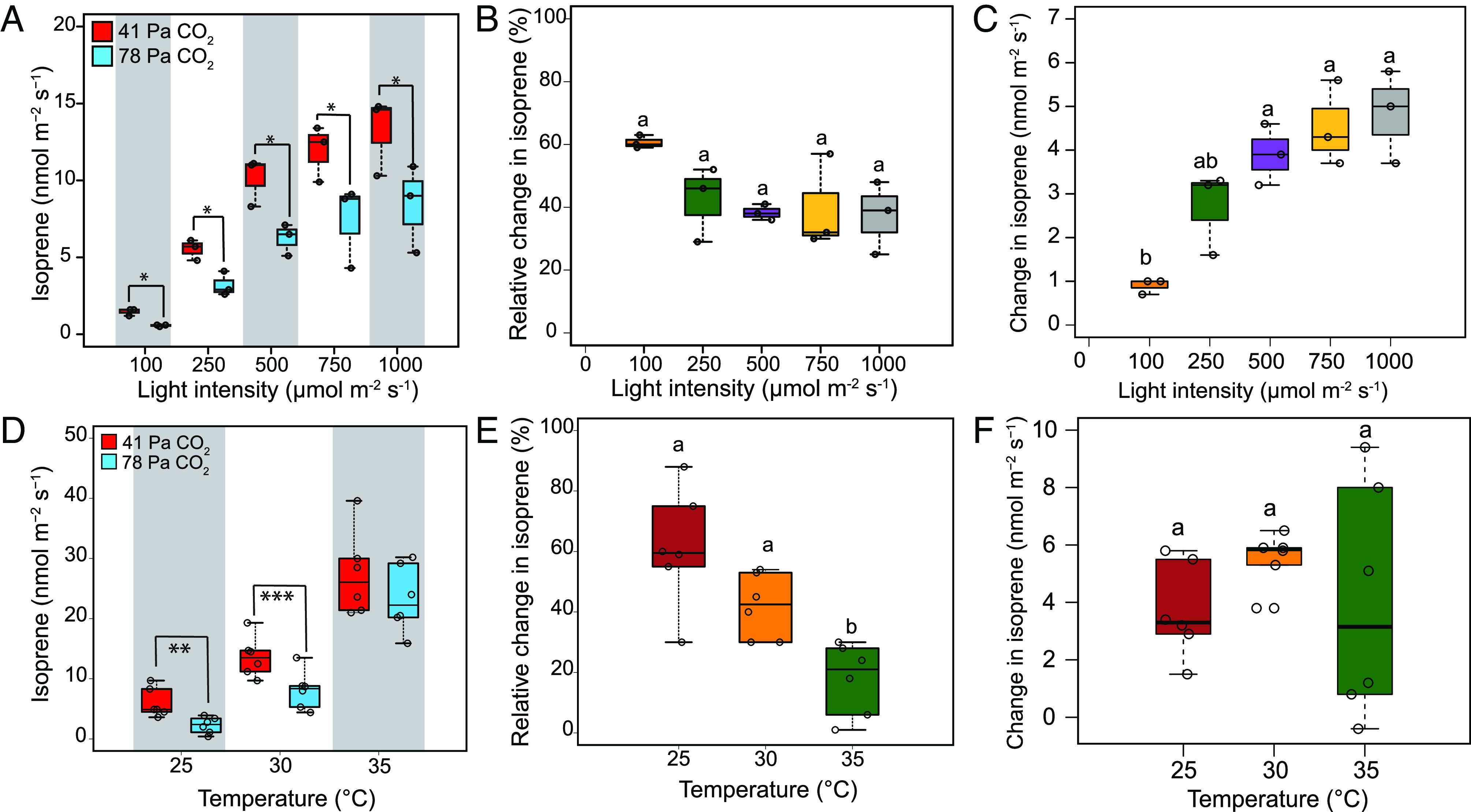
Effect of different light intensities and temperatures on high CO_2_-induced suppression of isoprene emission in poplar leaves. (*A*) Isoprene emission in poplar leaves (*n* = 3) at five different light intensities at 41 and 78 Pa CO_2_. Temperature was held constant at 30 °C. (*B*) Relative and (*C*) absolute changes in isoprene emission in poplar leaves (*n* = 3) at 41 Pa and 78 Pa CO_2_ under different light intensities. (*D*) Isoprene emission in poplar leaves (*n* = 6) at three different temperatures at 41 Pa and 78 Pa CO_2_. (*E*) Relative and, (*F*) absolute change in isoprene emission in poplar leaves (*n* = 6) between ambient and high CO_2_ at different temperatures. Light was held constant at 1,000 μmol m^−2^ s^−1^. Asterisks indicate significant decline in isoprene emission at 78 Pa CO_2_ compared with 41 Pa CO_2_ (*P < 0.05, **P < 0.01, and ***P < 0.001; Student’s two-tailed *t* test) in (*A* and *D*). Statistically significant differences by ANOVA and Tukey’s HSD (*P* < 0.001) are indicated by lowercase letters in *B*, *C*, *E*, and *F*. Whiskers of the box plots represent 95% CI.

Measurements of CO_2_-mediated inhibition of isoprene emission at different light levels were conducted in the same leaf after equilibrating the leaf at 41 Pa CO_2_. The decline of isoprene emission at high CO_2_ was significant at each light level (Fig. 2*A*), and the relative decrease in isoprene emission at high CO_2_ was similar at different light intensities (Fig. 2*B*). The absolute change in isoprene emission between 41 and 78 Pa CO_2_ increased with increasing light levels and showed significant difference between 100 μmol m^−2^ s^−1^ and 1,000 μmol m^−2^ s^−1^ light intensities (Fig. 2*C*). Assimilation rates increased significantly at 78 Pa CO_2_ at each light level; however, the increase was significantly lower at 100 μmol m^−2^ s^−1^ compared with 1,000 μmol m^−2^ s^−1^ light intensity (*SI Appendix*, Fig. S2*A*). The fraction of carbon lost as isoprene was also significantly lower at 78 Pa CO_2_ compared with 41 Pa CO_2_ at each light level (*SI Appendix*, Fig. S2*B*).

### Effect of Temperature on High CO_2_-Mediated Suppression of Isoprene Emission.

Measurements of change in isoprene emission between 41 and 78 Pa CO_2_ were conducted at 25 °C, 30 °C, and 35 °C. The high CO_2_-mediated decline in isoprene emission was significant at 25 °C and 30 °C, but not at 35 °C (Fig. 2*D*). Isoprene emission decreased by 61 ± 20% at 25 °C and 42 ± 11% at 30 °C when CO_2_ partial pressure was increased from 41 Pa to 78 Pa (Fig. 2*E*). However, the decrease of isoprene emission at 35 °C under 78 Pa CO_2_ was less (18 ± 12%), and it was significantly lower than that observed at 25 °C and 30 °C (Fig. 2*E*). The absolute change in isoprene emission was not significantly different at different temperatures (Fig. 2*F*). Although assimilation rates increased with increase in CO_2_ partial pressure, significant difference was not observed in the relative increase of assimilation rates at different temperatures (*SI Appendix*, Fig. S2*C*). However, the fraction of carbon lost as isoprene was significantly reduced at 78 Pa CO_2_ at each temperature (*SI Appendix*, Fig. S2*D*). Temperature coefficients (Q_10_) for isoprene emission and assimilation were calculated at 41 Pa and 78 Pa CO_2_ (Table 1)_._ The Q_10_ value for isoprene emission was 4.6 at 41 Pa CO_2_ and 10.3 at 78 Pa CO_2_ compared to the Q_10_ value of 1.2 for CO_2_ assimilation. Therefore, Q_10_ values for isoprene emission were higher than CO_2_ assimilation, more so at high CO_2_.

**Table 1.** Temperature sensitivity of isoprene emission and photosynthesis

CO_2_ (Pa)	Q_10_ isoprene	Q_10_ photosynthesis
41	4.6	1.2
78	10.3	1.2

Q_10_ was calculated using the equation: $Q_{10}={\left(\frac{R2}{R1}\right)}^{\left(10/(T2-T1)\right)}$ , where T2 = 35 °C, T1 = 25 °C, and R2 and R1 are the rates of isoprene emission or assimilation measured at 35 °C and 25 °C, respectively.

### Comparison of MEP Pathway Metabolite Levels at 41 and 78 Pa CO_2_.

**Fig. 3.**
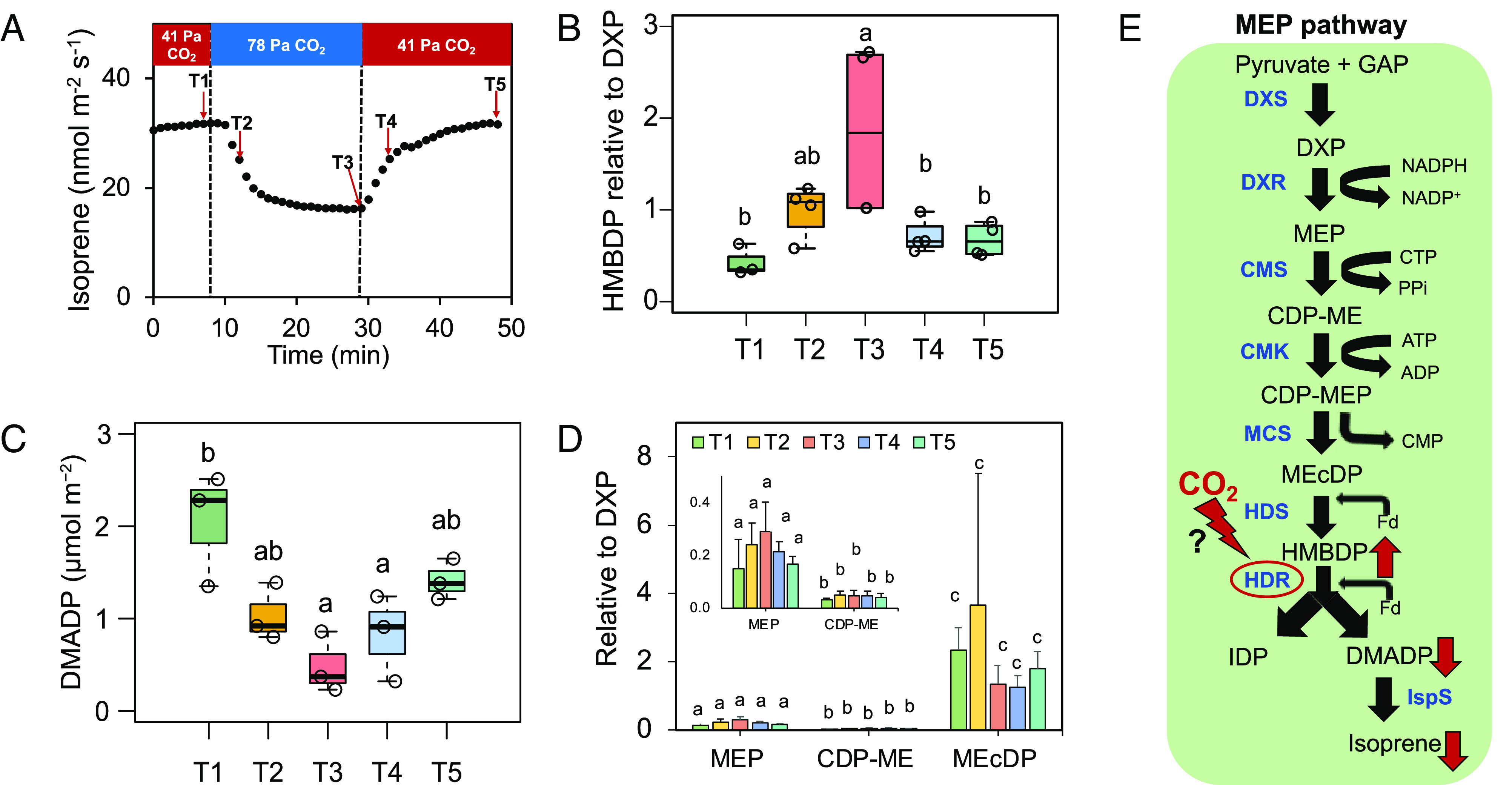
Change in the levels of MEP pathway metabolites at high CO_2_. (*A*) Plot showing time points on the isoprene emission curve where leaf samples were harvested. Levels of (*B*) HMBDP relative to DXP, (*C*) DMADP, and (*D*) other MEP pathway metabolites (MEP, CDP-ME, and MEcDP) measured in poplar leaves (*n* = 3 to 4) at 41 Pa and 78 Pa CO_2_ levels. Statistically significant differences by ANOVA and Tukey’s HSD (*P* < 0.05) are indicated by lowercase letters. (*E*) Schematic representation of isoprene biosynthesis via MEP pathway in chloroplasts and proposed regulatory point of isoprene suppression in high CO_2_. Whiskers of the box plots represent 95% CI. Bar plots represent mean ± SD for each group. Abbreviations: DXP = 1-deoxy-d-xylulose-5-phosphate, MEP = methylerythritol 4-phosphate, CDP-ME = 4-(cytidine-5′-diphospho)-2-C-methyl-D-erythritol, MEcDP = 2-C-methyl-d-erythritol-2,4-cyclodiphosphate, HMBDP = 4-hydroxy-3-methylbut-2-enyl-diphosphate, IDP = isopentenyl diphosphate, DMADP = dimethylallyl diphosphate, DXS = 1-deoxy-D-xylulose-5-phosphate synthase, DXR = 1-deoxy-D-xylulose-5-phosphate reductoisomerase, CMS = 4-diphosphocytidyl-2-C-methylerythritol synthase, CMK = 4-(cytidine-50-diphospho)-2-C-methyl-D-erythritol kinase, MCS = 2-C-methyl-Derythritol-2,4-cyclodiphosphate synthase, HDS = 4-hydroxy-3-methylbut-2-enyl-diphosphate synthase, HDR = 4-hydroxy-3-methylbut-2-enyl-diphosphate reductase, IspS = isoprene synthase.

Levels of DXP, MEP, 4-(cytidine-5′-diphospho)-2-C-methyl-D-erythritol (CDP-ME), 2-C-methyl-d-erythritol-2,4-cyclodiphosphate (MEcDP), and 4-hydroxy-3-methylbut-2-enyl diphosphate (HMBDP) were quantified using LC-MS/MS in leaf samples collected at the different time points of the isoprene emission curve (Fig. 3*A*). The level of HMBDP, normalized to DXP, was significantly higher at high CO_2_ (Fig. 3*B*)_._ HMBDP accumulated at T3, and then, it decreased at T5 upon returning to 41 Pa CO_2_. Since peaks of DMADP were not clearly detectable by LC-MS/MS, the in vivo pool size of DMADP was measured by integrating the isoprene emission after turning off the lights. As isoprene emission declined 3 min after switching to high CO_2_, the DMADP level also started to decrease at T2 and was significantly decreased at T3 compared to T1, when isoprene emission reached a steady minimum at high CO_2_ (Fig. 3*C*). Upon reexposure to 41 Pa CO_2_, DMADP increased fourfold relative to T3. There was no significant difference in the quantities of other metabolites between 41 and 78 Pa CO_2_ (Fig. 3*D*).

### Relationship between High CO_2_-Mediated Suppression of Isoprene Emission and Stomatal Signaling.

**Fig. 4.**
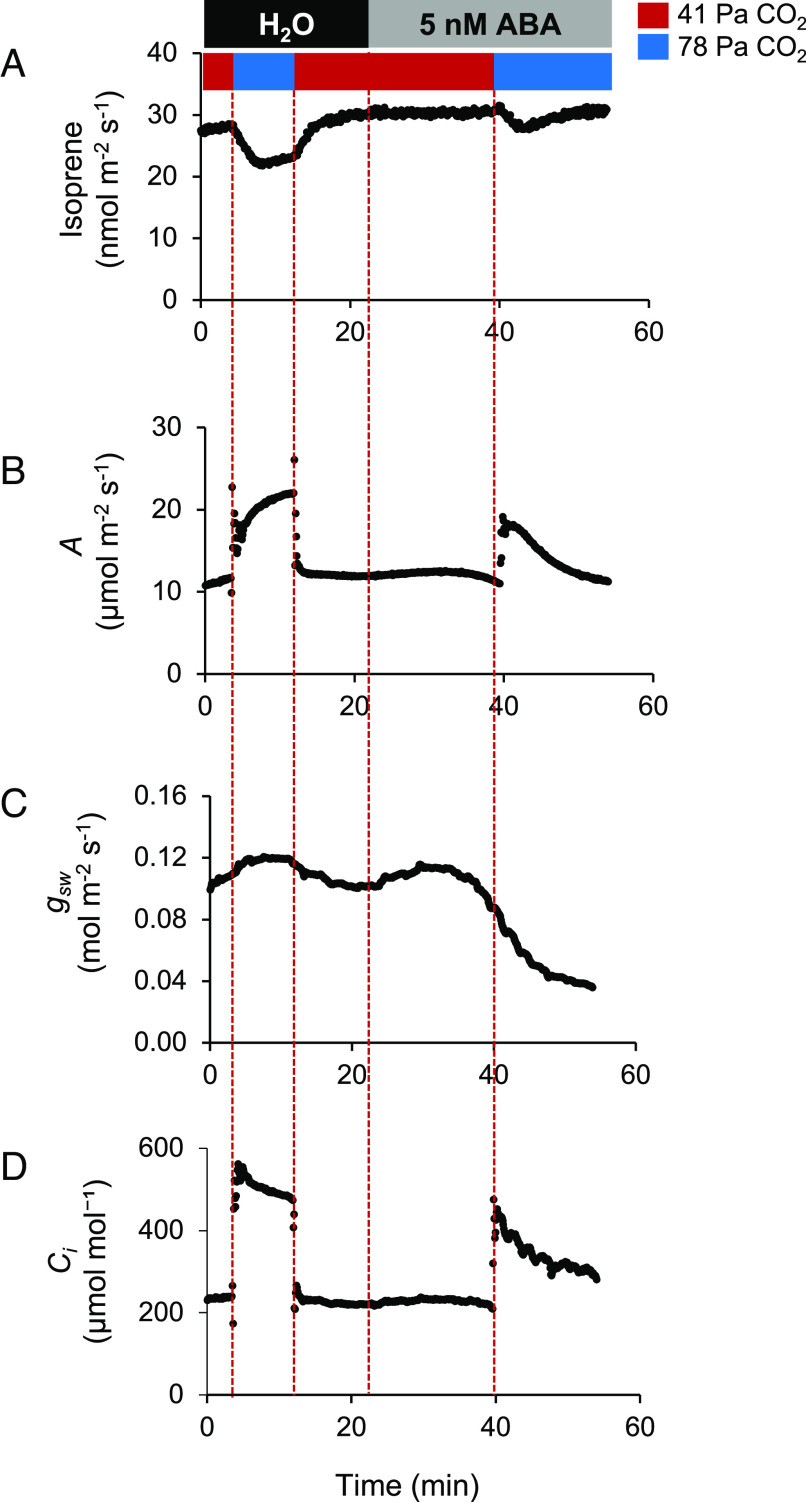
Relationship between high CO_2_-mediated changes of isoprene emission and ABA-dependent stomatal signaling pathway. Absolute change in (*A*) isoprene emission; (*B*) photosynthesis (*A*); (*C*) stomatal conductance (*g_sw_*); and (*D*) intercellular CO_2_ concentration (*C_i_*) measured in a poplar leaf at 41 Pa CO_2_ and 78 Pa CO_2_ levels in the presence of water (control) followed by 5 nM ABA treatment.

A detached leaf was first fed with water at 41 Pa CO_2_ followed by measuring isoprene emission at 78 Pa CO_2_ and then 41 Pa CO_2_. After isoprene emission stabilized, we fed the leaves with 5 nM ABA and found that there was no change in isoprene emission at 41 Pa CO_2_ after feeding the leaves with ABA (Fig. 4*A*), although stomatal conductance was declined by 11% of its initial value after 15 to 17 min of ABA feeding (Fig. 4*C*). Then, the CO_2_ partial pressure was increased to 78 Pa. Isoprene emission transiently declined, and photosynthesis increased at 78 Pa CO2 (Fig. 4*B*). However, stomatal closure due to ABA eventually resulted in decrease in *C_i_*(Fig. 4*D*) with a concomitant increase in isoprene (Fig. 4*A*).

### Effect of High CO_2_ on the H_2_O_2_ Level.

Since the [4Fe-4S] cluster of HMBDP reductase (HDR) is susceptible to reactive oxygen species, we quantified H_2_O_2_ levels in poplar leaves exposed to 41 or 78 Pa CO_2_. Our results indicated a nonsignificant change in the H_2_O_2_ level between 41 and 78 Pa CO_2_ (*SI Appendix*, Fig. S5).

## Discussion

The decline in isoprene emission at elevated CO_2_ is independent of assimilation rates and varying light intensities; however, it is reduced at high temperature. Isoprene emission decreases at high CO_2_ because the DMADP level decreases, although the HMBDP level increases. Additionally, high CO_2_-mediated inhibition of isoprene emission is independent of the ABA-mediated stomatal signaling pathway. Therefore, our results suggest that high CO_2_ signal inhibits the activity of the HDR such that the conversion of HMBDP to DMADP is impeded, resulting in lower isoprene emission at high CO_2_ (Fig. 3*E*).

While CO_2_ responsiveness of isoprene emission varies among the species, we observed a 42% decrease in isoprene emission from poplar leaves at elevated CO_2_ (Fig. 1*B*), which corresponded to previous findings (31–33). Our data also indicate that CO_2_-mediated suppression of isoprene emission is independent of stomatal conductance (*SI Appendix*, Fig. S1*B*), consistent with the findings of Jones and Rasmussen (34). Although our experiments show the effect of high CO_2_ on isoprene emission for a short time period, multiple studies demonstrated that growing plants at high CO_2_ can lead to a similar effect on isoprene emission in some species, including *Populus deltoides*, *Populus tremuloides*, and *Phragmites* (17, 32, 35). On the contrary, some previous studies (30, 36) showed that there was no change in canopy-level isoprene emission at high CO_2_ when the differences in leaf area and biomass were taken into account.

Typically, isoprene biosynthesis relies on photosynthesis for its carbon supply (10, 37). Furthermore, it was observed that ribulose 1,5-bisphosphate (RuBP) and ATP levels decreased, whereas 3-phosphoglyceric acid and triose phosphate levels were increased with rising CO_2_ concentrations (38). However, the decline of isoprene emission at elevated CO_2_ is unrelated to assimilation rates as shown by our data (Fig. 1*A*). Previously, Lantz et al. (22) also showed that the decline of isoprene at high CO_2_ is independent of TPU limitation, and PSII, PSI, or ATP synthase energetics.

We further explored the impact of environmental conditions like light and temperature on high CO_2_-mediated suppression of isoprene emission (Fig. 2). It is evident from earlier studies that isoprene emission increases exponentially with increasing light intensity (11) and the leaves exposed to sunlight emit more isoprene than those in shade (39, 40). Although isoprene emission is light-dependent, it was not clear whether the illumination level has any impact on the suppression of isoprene emission at elevated CO_2_. Our result (Fig. 2 *A* and *B*) confirmed that the decline of isoprene emission at high CO_2_ was independent of the supply of reductive energy equivalents, including ATP and NADPH from the photosynthetic electron transport chain. Furthermore, our results demonstrated that the impact of high CO_2_ on isoprene emission was substantially reduced at high temperatures (Fig. 2 *D* and *E*), which is consistent with previous reports (22, 41, 42). We also showed that decline in isoprene emission at elevated CO_2_ was independent of CO_2_ assimilation rates since we did not observe any significant difference in relative change of CO_2_ assimilation at different temperatures (*SI Appendix*, Fig. S2*B*). Indeed, temperature response of isoprene is accomplished primarily by regulation of isoprene synthase rather than substrate supply (14, 43). It is interesting that temperature not only regulates absolute rates of isoprene emission (12, 40) but also affects change in isoprene levels at high CO_2_ by dampening the suppression of isoprene emission. Arneth et al. (44) predicted that CO_2_-mediated suppression of isoprene emission is strong enough to offset the increase in emission due to warming climate and increase in plant biomass. Land-use change and vegetation dynamics will also affect future global isoprene emission, and some investigators expect that isoprene emission will likely remain the same or decrease in the future (45), while others expect increased leaf area index to result in more isoprene emission in a future high CO_2_ world (33). However, Lantz et al. (22) estimated an increase in isoprene emission by the year 2100 based on an IPCC climate model (800 ppm CO_2_ and 33 °C). Similarly, our results (Fig. 2*E*) also indicate that isoprene emission will increase under the combined effect of high temperature and elevated CO_2_ though without factoring in land use changes and natural vegetation composition. Our study is also based on short-term effects of temperature and CO_2_ increase which may have a more severe impact on isoprene emission than compared to a gradual rise in temperature and CO_2_, like it is occurring globally. We also observed a remarkable difference in Q_10_ between isoprene emission and CO_2_ assimilation (Table 1), which confirms the sensitivity of isoprene to temperature as observed in earlier studies (13, 22). Moreover, Q_10_ of isoprene emission doubled at 78 Pa CO_2_ compared to 41 Pa CO_2_ because of the loss of CO_2_ responsiveness of isoprene emission at high temperature. However, regulation of enzyme activities under the combined effect of increased temperature and CO_2_ requires further investigation.

Previous studies demonstrated that isoprene emission is correlated with the in vivo pool size of DMADP (29, 46, 47). In fact, Niinemets et al. (31) showed that DMADP levels decrease at high CO_2_ in some species, resulting in reduction of isoprene emission. However, the reason behind the reduced level of DMADP at high CO_2_ was not identified. Our data indicate that high CO_2_ exposure led to an increase in the HMBDP level but a decrease in the DMADP pool in poplar leaves, suggesting a possible interruption of HDR activity. It could be due to changes in the intracellular environment that affects the [4Fe-4S] cluster of HDR, leading to reduction in HDR activity. One hypothesis that H_2_O_2_ accumulates at high CO_2_ was not supported by the data. Therefore, identifying the mechanism of HDR activity regulation at elevated CO_2_ remains a pertinent question for future research. A recent study reported the differences in the activities of HDR isoforms in regulation of isoprenoid biosynthesis in other systems (48). Niinemets et al. (31) showed that MEcDP levels were not affected by high CO_2_, which is in parallel to our observation. An increase in HMBDP at high CO_2_ may also inhibit the activity of DXS (49), which possibly contributed to the insignificant changes in the levels of the metabolites downstream of DXS (Fig. 3*D*). Thus, our results indicate that HDR activity is a major target of high CO_2_-mediated regulation of MEP pathway (Fig. 3*E*).

There were multiple observations that led us to examine whether the CO_2_ sensing mechanism of the stomatal guard cells plays a role in regulation of the MEP pathway in the mesophyll cells that leads to the suppression of isoprene emission at high CO_2_. First, changes in isoprene emission under varying CO_2_ concentrations were similar to the stomatal response (*SI Appendix*, Fig. S4). Next, calcium spikes are associated with stomatal responses; feeding ethylene glycol tetra acetate (EGTA), a Ca^2+^ chelator, to detached leaves, affects isoprene emission in response to wounding in velvet bean (50). Since stomatal closure at elevated CO_2_ requires an ABA-dependent signaling mechanism (51–53) and stomatal closure is accelerated in the presence of ABA and high CO_2_ simultaneously (54), we investigated the effect of ABA on the suppression of isoprene emission at high CO_2_ to test whether similar mechanisms exist in the mesophyll cells that regulate the MEP pathway. We hypothesized that if the ABA-dependent signaling pathway of stomatal closure at high CO_2_ also affected the MEP pathway, then feeding ABA to poplar leaves should exaggerate the CO_2_ responsiveness of isoprene emission. Our results (Fig. 4) indicate that the decrease of isoprene emission at high CO_2_ is independent of the ABA-dependent stomatal signaling pathway; rather, it is entirely regulated by *C_i_* which is consistent with a previous study (55). More information on the different modes of isoprene emission regulation will help developing a large-scale mechanistic model of isoprene emission.

In summary, our study identifies the regulatory point of the MEP pathway under elevated CO_2_. This knowledge can be incorporated into the development of predictive models that can more accurately estimate future isoprene emission levels. Thus, we will be able to assess the potential consequences of climate change on isoprene emission from plants and its effect on atmospheric chemistry, plant health, and the ability of plants to adapt to the changing environmental conditions.

## Materials and Methods

### Plant Growth.

Poplar “NM6” hybrid (*Populus nigra X maximowiczii*) plants were grown from stem cuttings provided by the Great Lakes Bioenergy Research Center (GLBRC). The plants were grown in 11-L pots containing Suremix soil (Michigan Grower Products, Galesburg, Michigan, USA) under a greenhouse setting (16 h photoperiod, mean light intensity12 mol m^−2^ d^−1^, and day/night temperature 33 °C/22 °C) (*SI Appendix*, Fig. S6). Plants were alternately watered with deionized water and half-strength Hoagland’s solution every day. Plants were brought from the greenhouse to the lab for conducting experiments. Trees were periodically cut back to provide continuously flushing branches.

### Gas Exchange Studies and Isoprene Measurement.

Gas exchange and isoprene emission measurements were recorded simultaneously using a LI-COR 6800 Portable Photosynthesis System (LI-COR Biosciences) and a Fast Isoprene Sensor (FIS; Hills Scientific), respectively (56–58). A recently fully expanded mature leaf was used. Exhaust air from the LI-COR 6800 was fed into the FIS for isoprene measurements. The flow rate in the LI-COR 6800 was set at 500 μmol s^−1^, and the FIS flow rate was set such that it drew sample air from the LI-COR 6800 at 600 standard cubic centimeters per min (420 μmol s^−1^). A 3.225 ppm isoprene standard was used for the FIS calibration. First, we determined the background signal by measuring isoprene levels in the air flowing from the empty LI-COR chamber. A leaf was then clamped into a 6 cm^2^ chamber and allowed to equilibrate under the following conditions: light intensity of 1,000 µmol m^−2^ s^−1^ (50% blue light and 50% red light), temperature of 30 °C, CO_2_ of 420 µmol mol^−1^ (gases were mixed at different pressures and so are reported as mole fractions here), and water vapor content of 22 to 26 mmol mol^−1^ depending on laboratory room temperature. Measurements were logged every 5 s for both isoprene and gas exchange parameters.

### Harvesting Leaves for Metabolite Analysis.

Samples were harvested using an apparatus called Fast Kill freeze clamp that was built in-house and slightly modified from the version used by Li et al. (59) (*SI Appendix*, Fig. S3). The LI-COR 6800 head with a 6 cm × 6 cm chamber was mounted on the Fast Kill apparatus. We used cling film wrap to seal the top and bottom to create a closed chamber. Two gooseneck fiber optic illuminators were used to create a uniform field of illumination (1,000 µmol m^−2^ s^−1^). The leaf was clamped in the chamber and allowed to equilibrate under the conditions mentioned above. Leaf temperature was monitored with a thermocouple inserted into the chamber. When both isoprene and assimilation rates stabilized, two copper dies were cooled in liquid nitrogen and put on the apparatus, one above and one below the chamber. The leaf sample was smashed between these two dies. The time between light interruption and when the leaf sample was less than 0 °C was measured to be 35 ms. We harvested samples using the gas exchange chamber/freeze clamp at five time points of the isoprene emission curve (Fig. 3*A*) i) at 41 Pa CO_2_ after isoprene emission stabilized; T1 ii) 3 min after changing CO_2_ to 78 Pa; T2 iii) at 78 Pa CO_2_ after isoprene emission stabilized; T3 iv) 3 min after changing CO_2_ back to 41 Pa; T4 v) at 41 Pa CO_2_ after isoprene emission stabilized; T5. The samples were stored at −80 °C until further analysis.

### Extraction of Leaf Metabolites for LC-MS/MS.

Frozen leaf disks were ground into a fine powder in liquid nitrogen using mortar and pestle. Then, 500 μL of extraction buffer [3:1:1 acetonitrile: isopropanol: 20 mM ammonium bicarbonate (NH_4_HCO_3_) adjusted to pH 10 with ammonium hydroxide] was added to the ground plant material. They were then centrifuged at 14,000*g* for 10 min. The supernatant was then collected and aliquoted into glass inserts placed in 2 mL glass vials for LC-MS/MS analysis. Samples were analyzed by HPLC immediately after extraction.

### Metabolite Measurement by LC-MS/MS.

Standards of the following compounds DXP, MEP, CDP-ME, MEcDP, and HMBDP were purchased from Echelon Biosciences. These MEP pathway metabolites were separated using InfinityLab Poroshell 120 HILIC-Z, P column (2.1 × 100 mm, 2.7 micron with column ID) fitted on a Xevo TQ-XS mass spectrometer. Column temperature was set at 25 °C. Ammonium bicarbonate (20 mM, adjusted to pH 10.0 with ammonium hydroxide) and acetonitrile were used as mobile phase. A binary gradient was set up as described in *SI Appendix*, Table S1. Negative mode electrospray ionization was used. The following setup was used: capillary 1.00 kV, source temperature of 150 °C, and desolvation temperature of 400 °C.

### DMADP Measurement by Postillumination Isoprene Emission.

To quantify DMADP from postillumination isoprene emission, we followed the protocol as described by Rasulov et al. (60). To differentiate the system response from that of the plant, an isoprene standard of known concentration was injected into the empty leaf chamber using a needle; then, the needle was quickly removed to measure the decay kinetics of chamber clearing. Before recording measurements, a poplar leaf was equilibrated under the conditions mentioned above. The lights were turned off at the time points T1, T2, T3, T4, and T5 as described above. The difference of area under the curve with and without the plant normalized to the initial isoprene emission was calculated to determine the postillumination isoprene emission from the plant that represents the in vivo pool size of DMADP.

### ABA Feeding.

Detached leaves were used for the ABA-feeding experiment. The leaf was cut under water at the base of the petiole using a fresh razor blade. Then, it was immediately transferred into a test tube with water or 5 nM ABA for recording measurements, including isoprene emission and CO_2_ assimilation following the protocols described above.

### Quantification of the H_2_O_2_ Level.

H_2_O_2_ levels were quantified using the Amplex Red Assay kit (Amplex Red, dimethylsulfoxide, Horseradish peroxidase, and 5X phosphate buffer) purchased from Invitrogen (Thermo Fisher Scientific). Plant samples were ground in liquid nitrogen using a tissue homogenizer. Then, the powdered plant material was extracted in 5% trichloroacetic acid for 15 min and centrifuged at 14,000*g* for 10 min. The supernatant was neutralized with 2.1 M NH_4_HCO_3_. The extract (5.0 μL) was mixed with 45 μL 1× reaction buffer and 50 μL mix of 100 μM Amplex Red and 0.2 U/mL horseradish peroxidase. The mixture was incubated at room temperature for 30 min in the dark before recording its fluorescence on a microplate reader using 535 nm excitation and 595 nm emission filters. A standard curve was made using a series of H_2_O_2_ concentrations (0.1 μM, 0.2 μM, 0.4 μM, 0.8 μM, 1 μM, 2.5 μM, 5 μM, and 10 μM) to determine the levels of H_2_O_2_ in the leaf samples.

## Supplementary Material

Appendix 01 (PDF)

## Data Availability

All data are available at Dryad DOI: 10.5061/dryad.d7wm37q64 (61).
