# Supplementary material for: Hydroxymethylbutenyl diphosphate accumulation reveals MEP pathway regulation for high CO2-induced suppression of isoprene emission
Source: Proc Natl Acad Sci U S A. 2023 Oct 2;120(41):e2309536120. doi: 10.1073/pnas.2309536120 (PMC10576107; doi:10.1073/pnas.2309536120)
Supplement: Supplementary file 1 — Appendix 01 (PDF) [file pnas.2309536120.sapp.pdf]

Hydroxymethylbutenyl diphosphate accumulation reveals MEP pathway regulation for high CO_2-induced suppression of isoprene emission

Abira Sahu [a,b], Mohammad Golam Mostofa [a,b,c], Sarathi M. Weraduwage [a,b,c,d], and Thomas D. Sharkey [a,b,c,1]

[a] Department of Energy Plant Research Laboratory, Michigan State University, East Lansing 48824, Michigan, USA
[b] Plant Resilience Institute, Michigan State University, East Lansing, 48824, Michigan, USA
[c] Department of Biochemistry and Molecular Biology, Michigan State University, East Lansing, 48824, Michigan, USA
[d] Department of Biology and Biochemistry, Bishop's University, Sherbrooke, JIE0L3, Quebec, Canada

Supplementary materials

Fig. S1. Effect of high CO_2 on photosynthesis and stomatal conductance in poplar leaves.
Fig. S2. Effect of different light intensities and temperatures on photosynthesis and carbon cost of isoprene emission at high CO_2.
Fig. S3. Experimental setup for collecting leaf tissue for metabolite analysis.
Fig. S4. Effect of increasing CO_2 on isoprene emission and stomatal conductance (g_{sw}).
Fig. S5. H_2O_2 level in poplar leaves at ambient and high CO_2.
Fig. S6. Growth conditions (light and temperature) maintained in the greenhouse to grow poplar plants.
Table S1. Gradient table for binary solvents consisting of 20 mM NH_4HCO_3 in H_2O, pH~10 used for separation of MEP pathway metabolites.

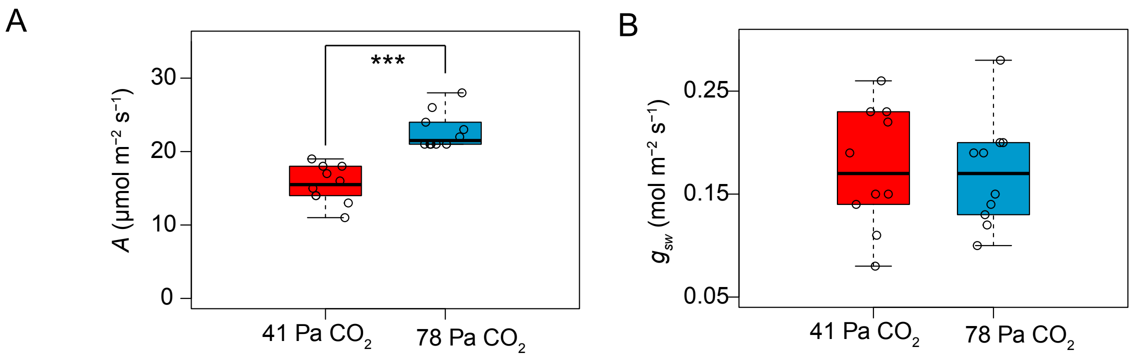

Fig. S1. Effect of high CO_2 on photosynthesis and stomatal conductance in poplar leaves. A, Photosynthesis (*A*); and B, stomatal conductance (g_{sw}) recorded in poplar leaves (n=10) after they reached a stable value at 41 Pa and 78 Pa CO_2. Asterisks indicate significant increase in photosynthesis at high CO_2 compared with ambient CO_2 ($P<0.001$; Student's t-test). Whiskers of the box plots represent 95% confidence interval.

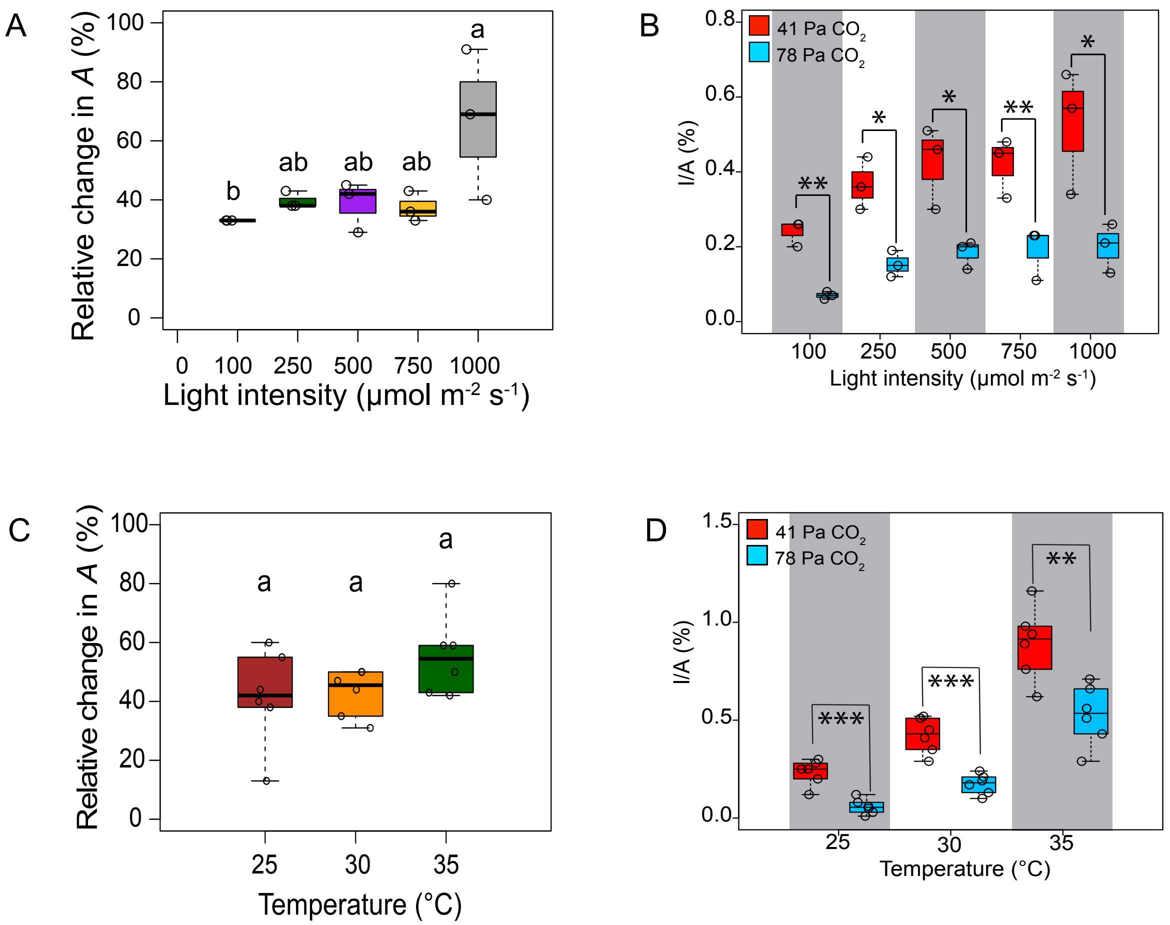

Fig. S2. Effect of different light intensities and temperatures on photosynthesis and carbon cost of isoprene emission at high CO_2. A, Relative change in photosynthesis and B, Carbon lost as isoprene as a fraction of net carbon assimilation between 41 Pa and 78 Pa CO_2 in poplar leaves (*n*=3) at different light intensities; and C, Relative change in photosynthesis and D, Carbon lost as isoprene as a fraction of net carbon assimilation between 41 Pa and 78 Pa CO_2 in poplar leaves (*n*=6) at different temperatures. Statistically significant differences by ANOVA and Tukey's HSD ($P<0.001$) are indicated by lowercase letters in A and C. Asterisks indicate significant decline in isoprene emission at 78 Pa CO_2 compared with 41 Pa CO_2 (* $P<0.05$, ** $P<0.01$, *** $P<0.001$; Student's two-tailed t-test) in B and D. Whiskers of the box plots represent 95% confidence interval.

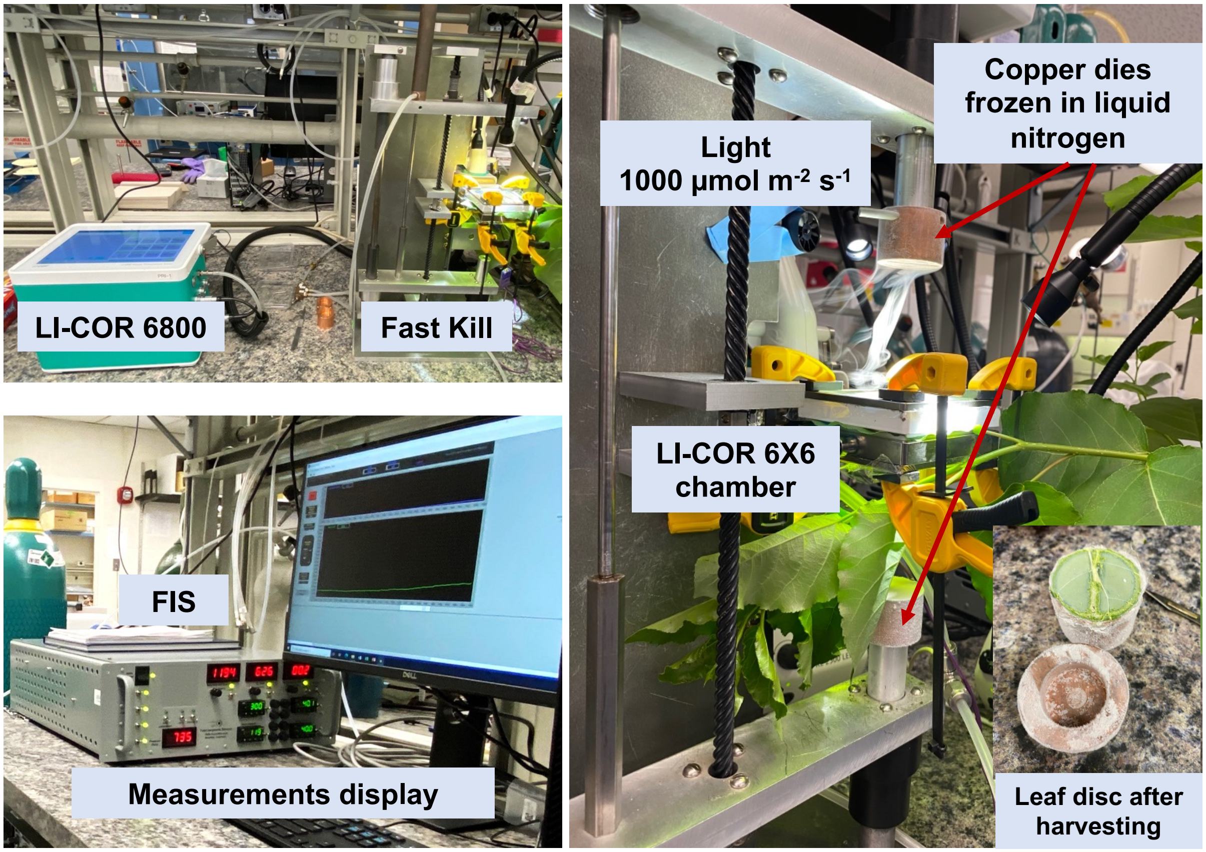

Fig. S3. Experimental setup for collecting leaf tissue for metabolite analysis. A LI-COR head attached to a 6 cm x 6 cm chamber is mounted on the Fast Kill apparatus. A leaf is clamped in the chamber and isoprene emission is monitored using the FIS while simultaneously recording photosynthetic measurements in the LI-COR 6800 console. Copper dies frozen in liquid nitrogen are used to flash freeze leaf discs and collect samples at specific time points.

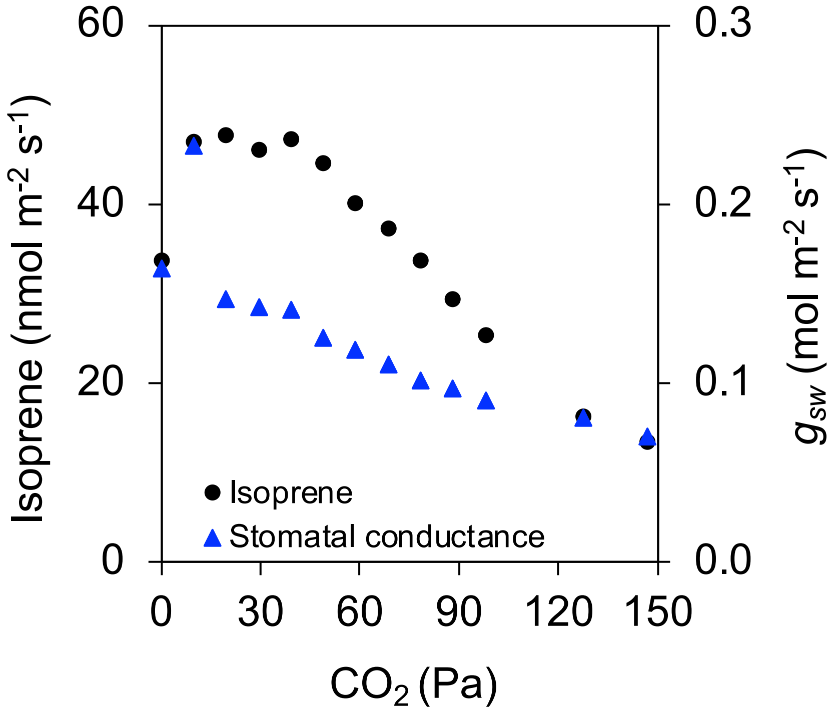

Fig. S4. Effect of increasing CO_2 on isoprene emission and stomatal conductance (g_{sw}).

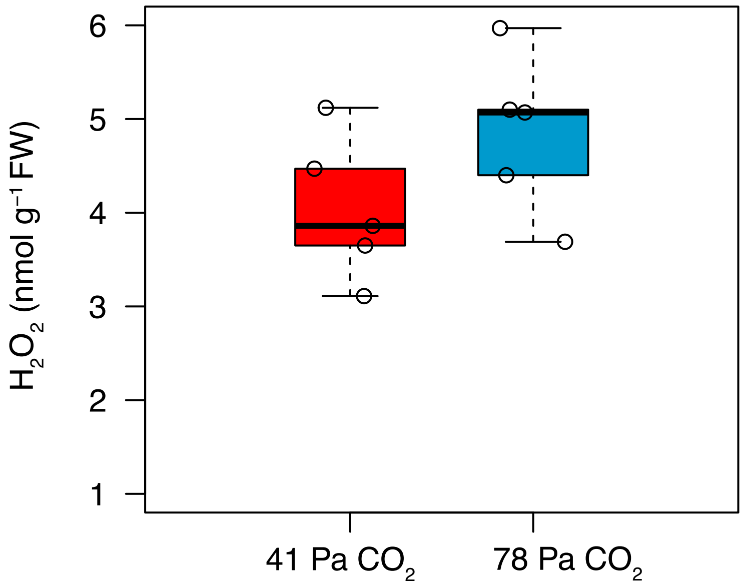

Fig. S5. H_2O_2 level in poplar leaves at ambient and high CO_2. No significant difference in H_2O_2 level was observed. Whiskers of the box plots represent 95% confidence interval.

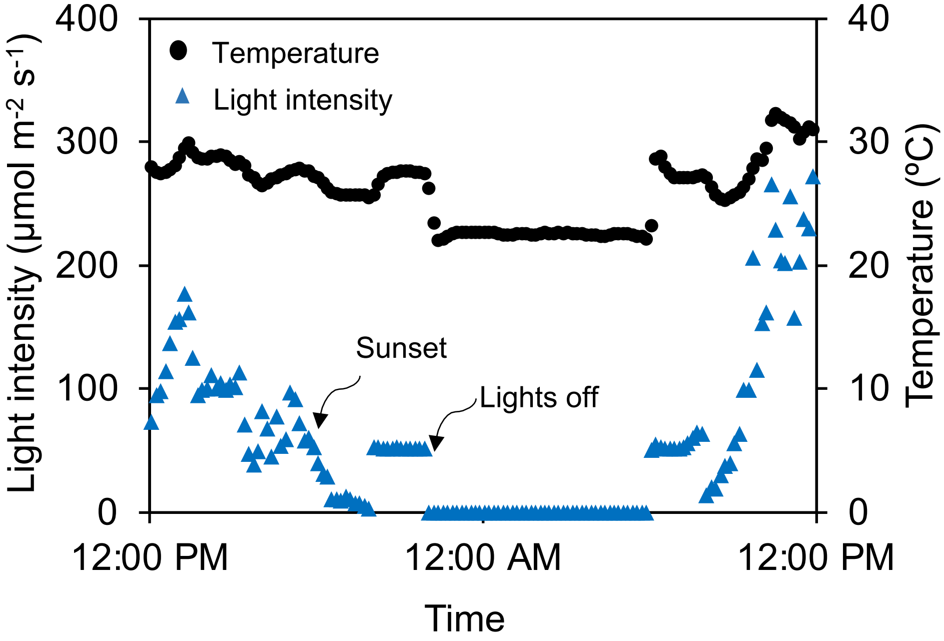

Fig. S6. Growth conditions (light and temperature) maintained in the greenhouse to grow poplar plants.

Time (min)	Flow Rate (mL/min)	%A	%B
0.00	0.2	20	80
2.00	0.2	20	80
6.00	0.2	60	40
8.00	0.2	60	40
8.10	0.2	20	80
10.00	0.2	20	80

A: 20mM NH_4HCO_3 in H_2O, pH~10
B: Acetonitrile

Table S1. Gradient table for binary solvents consisting of 20 mM NH_4HCO_3 in H_2O, pH~10 used for separation of MEP pathway metabolites.
